# Characteristics of Older Adults who Receive Assistance with Management of Multidrug Regimens

**DOI:** 10.1093/geroni/igab046.2782

**Published:** 2021-12-17

**Authors:** Rachel O'Conor, Julia Yoshino Benavente, Mogan Eifler, Lauren Opsasnick, Laura Curtis, Lee Lindquist, Michael Wolf

**Affiliations:** 1 Northwestern University, Chicago, Illinois, United States; 2 Northwestern University Feinberg School of Medicine, Chicago, Illinois, United States

## Abstract

Many older adults manage multiple chronic conditions requiring adherence to multidrug regimens, yet half are non-adherent, increasing their risk of hospitalization for poorly controlled chronic conditions. Few studies have investigated whether caregivers support medication-related behaviors of community-dwelling older adults. We interviewed 97 patient-caregiver dyads participating in a cognitive aging cohort study to identify factors associated with caregiver assistance in managing multidrug regimens. Patients completed a neuropsychological battery covering five cognitive domains. Health literacy and patient activation were measured using the Newest Vital Sign and Consumer Health Activation Index, respectively. Caregivers reported their medication-related involvement. Predictors of involvement in medication-related tasks were examined using logistic regression models. Patients were on average 71 years old, managing 4 comorbidities and prescribed 5 medications. The majority were female (73%) and identified as Black (46%) or White (47%). Caregivers’ mean age was 65 years; half were female (53%), were predominantly spouses (57%) or children (26%), and lived with the patient (61%). 31% of caregivers ordered patients’ prescribed medications, 40% helped manage their medications, and 50% spoke with the patient’s clinician about their clinical care. Cognitive impairment (OR 2.60, 95% CI 1.08-6.25), limited health literacy (OR 2.97, 95% CI 1.26-6.97), and ≥3 comorbidities (OR 2.14, 95% CI 1.06-9.30) were associated with medication management assistance. Patient activation, gender, cohabitation, or relationship were not associated. These findings suggest that caregivers are assisting with older adults’ medication management and should be included in clinical discussions about medication management, especially among patients with cognitive impairment, low health literacy or multimorbidities.

